# COVID-19 Vaccination and Rates of Infections, Hospitalizations, ICU Admissions, and Deaths in the European Economic Area during Autumn 2021 Wave of SARS-CoV-2

**DOI:** 10.3390/vaccines10030437

**Published:** 2022-03-12

**Authors:** Dominika Sikora, Piotr Rzymski

**Affiliations:** 1 Department of Environmental Medicine, Poznan University of Medical Sciences, 60-806 Poznań, Poland; dominika.sikora97@o2.pl; 2 Doctoral School, Poznan University of Medical Sciences, Fredry St. 10, 61-701 Poznań, Poland; 3 Integrated Science Association (ISA), Universal Scientific Education and Research Network (USERN), 60-806 Poznań, Poland

**Keywords:** pandemic, coronavirus, vaccine hesitancy, vaccine efficacy, Europe

## Abstract

The COVID-19 vaccination campaigns were met with a varying level of vaccine hesitancy in Europe. We analyzed the potential relationships between COVID-19 vaccine coverage in different countries of the European Economic Area and rates of infection, hospitalizations, admissions to intensive care units (ICU), and deaths during the autumn 2021 SARS-CoV-2 wave (September−December). Significant negative correlations between infection rates and the percentage of fully vaccinated individuals were found during September, October, and November, but not December. The loss of this protective effect in December is likely due to the emergence of the omicron (B.1.1.529) variant, better adapted to evade vaccine-induced humoral immunity. For every considered month, the negative linear associations between the vaccine coverage and mean number of hospitalizations (r= −0.61 to −0.88), the mean number of ICU admissions (r= −0.62 to −0.81), and death rate (r= −0.64 to −0.84) were observed. The results highlight that vaccines provided significant benefits during autumn 2021. The vaccination of unvaccinated individuals should remain the primary strategy to decrease the hospital overloads, severe consequences of COVID-19, and deaths.

## 1. Introduction

**Figure 1:**
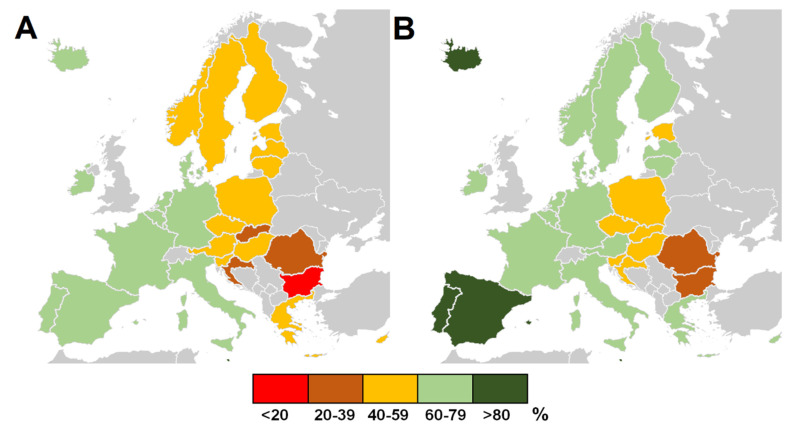
The share of fully vaccinated individuals in different EEA countries at the beginning of (**A**) September 2021 and (**B**) December 2021. Based on [19].

The COVID-19 pandemic has been met with an unprecedented and rapid scientific response resulting in the emergence of diagnostic methods and a better understanding of viral pathogenicity, immune response to the infection, and potential therapeutic targets [1]. Moreover, a great effort has been put forward to develop vaccine candidates using various approaches, including classical (inactivated, live-attenuated, recombinant vaccines) and more innovative (mRNA, DNA, adenoviral vector vaccines) ones. The COVID-19 vaccines were made available with unseen speed due to years of basic and applied research, technological advances and platforms that enable the rapid development of candidates (e.g., mRNA), significant funding, running multiple trials in parallel, and regulatory agencies working at an extraordinary pace [2]. Until the end of 2021, four COVID-19 vaccines were approved in the European Union: two mRNA vaccines, BNT162b2 (BioNTech/Pfizer, Germany, Mainz/New York, NY, USA) and mRNA-1273 (Moderna, Cambridge, MA, USA), given as two doses 21 and 28 days apart, respectively, as well as two adenoviral vector vaccines: AZD1222 (Oxford/AstraZeneca, UK/Sweden), administered as two doses 4–12 weeks apart, and Ad26.COV2.S Janssen/Johnson & Johnson, Leiden, Netherlands/New Brunswick, NJ, USA), given as a single dose. Over 750 million doses of mRNA vaccines and nearly 200 million doses of adenoviral vector vaccines were used in 2021 in the European Economic Area (EEA) [3]. The short-term results of pre-authorization clinical trials have shown that these vaccines have a high efficacy against symptomatic and severe infection [4,5,6,7]. However, the emergence of novel SARS-CoV-2 variants, characterized by increased transmissibility and partial evasion of antibody neutralization in vaccinated individuals, raised concerns about whether the vaccines will remain effective enough. This has mainly concerned the B.1.617.2 (delta) variant that was first identified in India in December 2020 and became dominant in the European Region by June/July 2021 [8]. A number of post-authorization studies originating from different countries in mid-2021 have shown that despite SARS-CoV-2 evolution, vaccination continues to decrease the risk of contracting the virus [2,9,10,11], while in the case of breakthrough infection, the risk of severe disease, hospitalization, and death is significantly reduced [12,13,14,15]. However, SARS-CoV-2, similarly to other respiratory viruses, reveals a strong seasonal pattern in the temperate zone, with infection rates rising in the autumn-winter season [16,17,18]. Various EEA countries entered this season in September 2021 with strikingly different vaccine coverage, ranging from 17.0% of fully vaccinated individuals in Bulgaria to 80.3% in Malta (Figure 1a) [19]. By December 2021, the difference in vaccine coverage was also apparent (Figure 1b). These discrepancies were driven not by a limited availability of doses but by differences in vaccine hesitancy levels and approaches to promoting COVID-19 vaccinations [20,21,22]. In this study, we analyzed whether vaccine coverage in the EEA countries was related to the four pandemic indices during the autumn 2021 SARS-CoV-2 wave (September–December), i.e., rates of infection, hospitalizations, ICU admission, and death due to COVID-19.

## 2. Materials and Methods

To test whether the vaccination rates had an effect on pandemic indices in EEA during the autumn 2021 SARS-CoV-2 wave (September–December), we have retrieved Centre for Disease Prevention and Control (ECDC) data [23,24] on SARS-CoV-2 infections, hospitalizations, admission to intensive care units (ICU), and deaths due to COVID-19. These data were used to calculate the monthly infection rate and death rate per 100 thousand inhabitants of each country and the mean daily number of hospitalized patients and patients admitted to ICU per 100 thousand inhabitants (hospitalization/ICU admission rate was not calculated to avoid counting the same patient multiple times as ECDC data show the total number of patients in hospitals/ICU for each day of the month). Data on vaccination rates for each country were retrieved from Our World in Data [19]. To this end, the percentage of fully vaccinated individuals (who received two doses of BNT162, mRNA-1273 or AZD1222, or a single dose of Ad26.COV2.S) in the population on the first day of each month was used (or if not provided, the nearest day for which the data was available). To analyze the relationship between the pandemic indices and vaccination rate for each month and the entire autumn 2021 wave (September−December), the Pearson correlation coefficient (r) was employed.

## 3. Results

**Figure 2:**
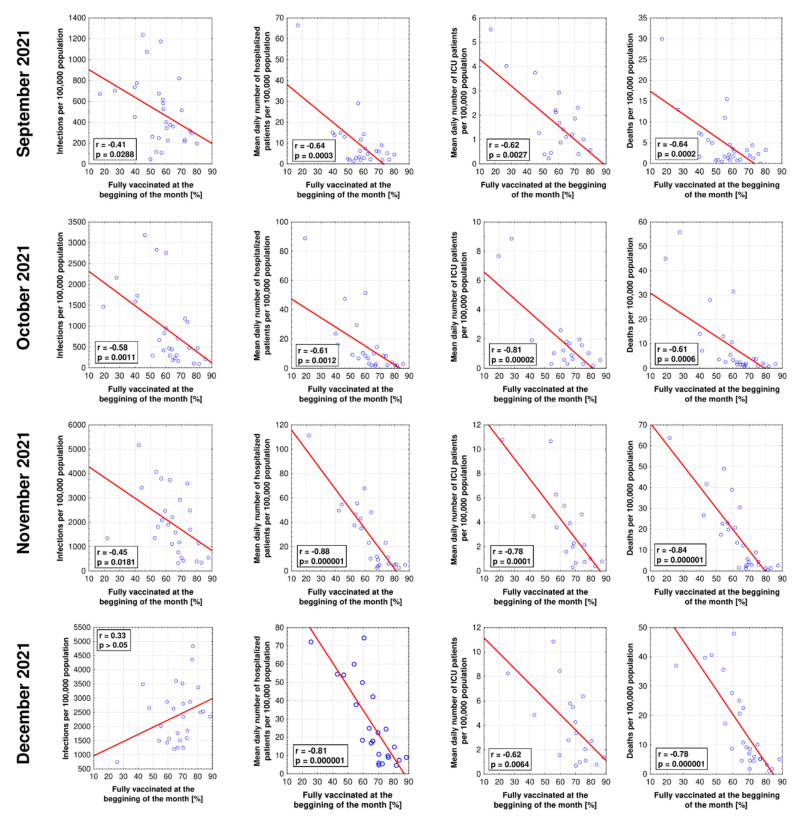
The correlations between the share of fully vaccinated individuals and the infection rate, mean daily hospitalizations and ICU admissions, and death rate in the autumn 2021 SARS-CoV-2 wave in EEA countries.

As demonstrated in Figure 2, most pandemic indices during the considered months were lower in better-vaccinated populations of EEA. Significant negative correlations between the infection rate and vaccine coverage were found in September, October, and November 2021, with Pearson r ranging from −0.41 to −0.58. For December, this correlation was statistically insignificant. In all four considered months, the vaccination rate was negatively associated with reduced patient overloads at hospitals, including a lower mean number of hospitalized patients per 100,000 population and mean number of patients requiring admission to ICU. Furthermore, the vaccination rate at the beginning of each month was negatively correlated with the rate of deaths due to COVID-19. Particularly strong correlations were found during November and December 2021, with Pearson r values of −0.84 and −0.74, respectively (Figure 2). 

## 4. Discussion

The results presented in this paper add to the body of knowledge on COVID-19 vaccine effectiveness. They show that populations with higher vaccination rates revealed better preparedness for the autumn 2021 wave despite the emergence of more transmissible SARS-CoV-2 variants and favorable environmental conditions for viral spread. This contradicts the frequent conviction of anti-vaccine groups that vaccines would not pass the test during this period. Notably, until November, vaccination has been related to lower infection rates—this effect was, however not seen in December 2021. This may be due to the B.1.1.529 (omicron) variant, first identified in Botswana and South Africa in November 2021. Although it started to rapidly spread worldwide, at the end of November the delta variant was still dominant in Europe, while the contribution of omicron to the infections was negligible; its highest share was reported by Austria—2.3% [19]. According to the ECDC data, it became increasingly frequent from the beginning of December, particularly in some countries with excellent COVID-19 vaccine coverage, such as Portugal and Spain. It was already a dominant variant in these regions in the second half of the month [25]. Omicron is highly transmissible and more effective at evading the antibodies than the delta variant, altogether increasing the risk of reinfections and breakthrough infections [26,27], possibly leading to the loss of the protective effect of vaccines. In addition, there is evidence that protection against disease gradually declines as the serum antibody level decreases [28,29,30]. Importantly though, the humoral immune response against the omicron variant can be restored by administration of booster doses [31,32].

However, throughout the considered period, hospitalizations, ICU admissions, and deaths due to COVID-19 were strongly negatively correlated with vaccination rates. This clearly shows that during the autumn 2021 wave, they met the primary goal of vaccinology, which has always been to decrease the clinical severity of infection [33,34]. Studies have shown that variants such as delta and omicron are recognized by components of cellular immunity that play a vital role in virus elimination and a decreased risk of severe disease during a breakthrough infection [35,36]. These findings strongly advocate that vaccinating unvaccinated individuals should remain the priority, including in low-income countries outside EEA that suffer from vaccine inequity [34]. Less vaccinated European countries must consider changes in their previous strategies to promote vaccination to overcome vaccine hesitancy, which in 2021 was particularly apparent in Central and Eastern Europe. 

One should note the study limitations. The correlations evidenced here do not necessarily imply a causal link. Although numerous clinical and observational studies provide evidence for the efficacy of COVID-19 vaccines, the non-pharmaceutical interventions, adherence to sanitary recommendations, and testing strategies implemented by the governments could also influence the investigated parameters, particularly infection rates. Similarly, the death rates may not solely be influenced by vaccination status but also the efficiency and capacity of the health care system, which may vary across countries. 

## 5. Conclusions

In conclusion, the present study provides additional evidence that COVID-19 vaccination is the most rational decision under pandemic conditions. In general, better vaccinated populations of EEA displayed lower rates of infections, hospitalizations, ICU admissions, and deaths due to COVID-19 during the autumn 2021 wave of SARS-CoV-2. However, the relationship with the infection rate was lost in December, likely due to the emergence and rapid spread of the omicron variant; the presented data show the positive effect of vaccinations on reduced disease severity. 

## Data Availability

The data presented in this study are available from the corresponding author on reasonable request.
